# L-Type Calcium Channels Do Not Play a Critical Role in Chest Blow Induced Ventricular Fibrillation: Commotio Cordis

**DOI:** 10.1155/2016/5191683

**Published:** 2016-01-26

**Authors:** Christopher Madias, Ann C. Garlitski, John Kalin, Mark S. Link

**Affiliations:** ^1^Cardiac Electrophysiology Service, Section of Cardiology, Rush University Medical Center, Chicago, IL, USA; ^2^Cardiac Arrhythmia Center, Division of Cardiology, Tufts Medical Center (TMC), P.O. Box 197, 800 Washington Street, Boston, MA 02111, USA

## Abstract

*Background*. In a commotio cordis swine model, ventricular fibrillation (VF) can be induced by a ball blow to the chest believed secondary to activation of mechanosensitive ion channels. The purpose of the current study is to evaluate whether stretch induced activation of the L-type calcium channel may cause intracellular calcium overload and underlie the VF in commotio cordis.* Method and Results*. Anesthetized juvenile swine received 6 chest wall strikes with a 17.9 m/s lacrosse ball timed to the vulnerable period for VF induction. Animals were randomized to IV verapamil (*n* = 6) or placebo (*n* = 6). There was no difference in the observed frequency of VF between verapamil (19/26: 73%) and placebo (20/36: 56%) treated animals (*p* = 0.16). There was also no significant difference in the combined endpoint of VF or nonsustained VF (21/26: 81% in verapamil versus 24/36: 67% in controls, *p* = 0.22).* Conclusions*. In this experimental model of commotio cordis, verapamil did not prevent VF induction. Thus, in commotio cordis it is unlikely that stretch activation of the L-type calcium channel with resultant intracellular calcium overload plays a prominent role.

## 1. Introduction

Sudden cardiac death due to the induction of ventricular fibrillation (VF) by blunt chest wall blows is defined as commotio cordis. Most commonly occurring in the setting of sport, commotio cordis has been reported with increasing frequency [1, 2]. Commotio cordis is a primary electrical event in which both the timing of impact and a rapid rise in left ventricular (LV) pressure are critical [3–6]. The rapid rise in LV pressure likely results in myocardial cell membrane stretch and deformation of the cell membrane with resultant activation of mechanosensitive ion channels. The K^+^_ATP_ channel is among the stretch sensitive ion channels that have been implicated in the pathophysiology of commotio cordis [7].

**Figure 1:**
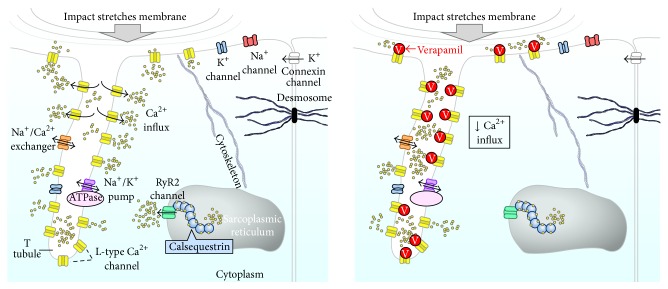
Impact of the chest wall marked increases left ventricular intracavitary pressure which in turn amplifies left ventricular wall strain and myocyte membrane stretch. This cell membrane deformation may activate stretch activated ion channels, which may in part cause the ventricular fibrillation in commotio cordis. In the left panel, a placebo animal, there is an increased influx of calcium due to stretch activation of the I_Ca,L_ channel. If the I_Ca,L_ channel is involved in commotio cordis, blockade of the channel (right panel) should reduce the ventricular fibrillation seen in our model.

The L-type calcium channel (I_Ca,L_) may also be activated by cell membrane stretch with resultant calcium influx into the cell (Figure 1) [8, 9]. Intracellular calcium overload underlies the mechanism of VF in catecholaminergic polymorphic ventricular tachycardia (CPVT). Intracellular calcium overload may also contribute to arrhythmogenesis in the Long QT Syndrome (LQTS), particularly in the Timothy syndrome [10].

In the current experiment we test the hypothesis that chest wall impact activates I_Ca,L_, causing an intracellular calcium spike, which can result in early-after-depolarizations (EADs) or delayed-after-depolarizations (DADs) and induce VF. If this is the case, then blocking I_Ca,L_ prior to chest wall impact should reduce the incidence of VF in commotio cordis. Verapamil, a synthetic papaverine derivative, primarily blocks I_Ca,L_ but also may have some blocking effect on the ryanodine receptor, both of which may contribute to intracellular calcium overload.

## 2. Methods

In conformity with the regulations of the Association for Assessment and Accreditation of Laboratory Animal Care, the Institutional Animal Care and Use Committee of the Tufts Medical Center (Boston, MA, USA) approved the research protocol. Twelve male domesticated swine with a weight from 15 to 20 kg were anesthetized with ketamine and inhaled isoflurane. Animals were intubated and placed on a respirator, and general anesthesia was maintained throughout the experiment with isoflurane (2% isoflurane gas in 100% oxygen gas). Millar Mikrotip^*®*^ (Houston, TX, USA) pressure catheters were introduced into the left ventricle via the femoral artery. Electrocardiographic tracings and left ventricular pressures were recorded continuously utilizing an analogue-to-digital converter (Chart^*®*^ software; AD Instruments, Mountain View, CA, USA). Recordings were sampled at 2,000 Hz, not filtered, and saved on a laptop computer.

Each animal was placed prone in a sling to approximate physiologic cardiac anatomy and hemodynamics. Chest blows were directed toward the anatomic center of the left ventricle as identified by transthoracic echocardiography. Chest blows were delivered by a lacrosse ball propelled at 17.9 m/s. Speed of the impact object was assessed using a chronograph (Oehler Research, Austin, TX, USA) modified for low velocities. Triggered from the surface electrocardiograms of the swine, the release and subsequent impact of the ball were gated to the cardiac cycle using a commercially available cardiac stimulator (EP-2, EP Medical, Inc., Budd Lake, NJ, USA). All impacts were timed to the vulnerable window for VF induction in commotio cordis (10 to 40 msec prior to the peak of the T-wave) [3].

Prior to impact, swine were randomly assigned to either calcium channel blockade with a verapamil infusion (0.4 mg/kg) or placebo (normal saline, 100 ml) [11]. Five minutes prior to the first chest wall impact, a technician not involved in the subsequent portions of the study administered the solutions intravenously. Investigators performing the chest blows remained blinded to the solutions (verapamil or control) each animal received. Measurements and interpretations of all electrocardiographic data were performed by a single investigator blinded to the solution administered. Animals given verapamil had a similar heart rate (106 ± 23 versus 108 ± 13 bpm; *p* = 0.89) compared to control animals but had a lower left ventricular systolic pressure (63 ± 16 versus 89 ± 12 mmHg; *p* = 0.01).

Ventricular fibrillation was defined as polymorphic ventricular arrhythmia requiring defibrillation. Nonsustained VF was defined as ≥3 beats of VF that terminated spontaneously. If VF occurred after a chest blow, the animal was immediately defibrillated. After each episode of VF, the animal's blood pressure, heart rate, and left ventricular ejection fraction (by echocardiography) were monitored. If these parameters returned to the baseline levels, repeat impacts were delivered for a total of 6 impacts per animal.

Continuous data were reported as mean ± SD. Differences between the groups were analyzed by chi-square (or Fisher's exact test, where appropriate), linear regression for continuous outcome variables, and logistic regression for dichotomous outcome variables. Analysis was performed in SAS statistical software (Version 8, Cary, NC, USA).

## 3. Results

**Figure 2:**
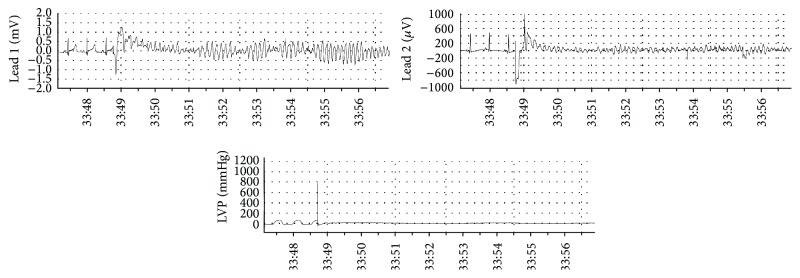
Initiation of ventricular fibrillation with a chest wall impact of a 40 mph lacrosse ball directly over the cardiac silhouette of an 18 kg swine. The impact occurs on the upslope of the T-wave and produces an immediate left ventricular pressure (LVP) rise to 800 mmHg and ventricular fibrillation.

**Figure 3:**
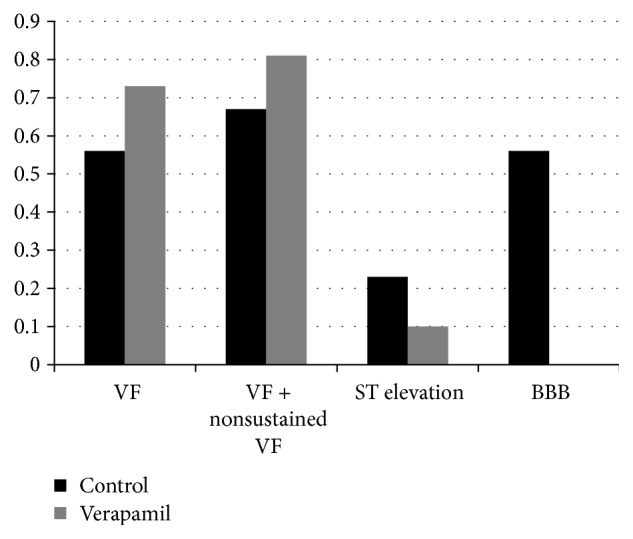
Bar graph of the results of 62 impacts to 12 animals with 36 impacts in 6 control animals and 26 impacts in 6 animals given verapamil. There was no difference in the endpoints of ventricular fibrillation (VF) or the combined endpoint of VF + nonsustained VF. Verapamil administration did decrease the severity of ST segment elevation and the induction of a bundle branch block (BBB).

Twelve domesticated swine received a total of 62 impacts within the vulnerable time window for VF induction. Initiation of VF was observed in 19 of 26 (73%) impacts in animals given verapamil, compared with 20 of 36 (56%) in control animals (*p* = 0.16) (Figures 2 and 3). Ventricular fibrillation occurred immediately following the chest blow. Despite defibrillation, two animals in the verapamil group could not be resuscitated after induction of VF from the initial chest impact. Defibrillation terminated all other VF episodes with normalization of blood pressure and LV systolic function.

Nonsustained VF was observed in 2 of 7 (28%) verapamil strikes compared with 4 of 16 (25%) controls. Thus, the combined endpoint of VF or nonsustained VF was met in 21 of 26 (81%) verapamil strikes compared with 24 of 36 (67%) controls (*p* = 0.22).

In strikes that did not induce VF, the frequency of categorical ST elevation did not differ between groups. However, pretreatment with verapamil did result in a diminished magnitude of ST elevation (100 ± 65 mV in the verapamil animals compared with 233 ± 105 mV in the controls; *p* = 0.01). Verapamil also reduced the frequency of chest blow induced bundle branch block (BBB), 0 of 7 verapamil strikes compared to 9 of 16 (56%) placebo strikes (*p* = 0.03). Peak left ventricular pressure after impact was slightly greater for animals pretreated with verapamil. Impact in verapamil animals caused a mean left ventricular peak pressure of 689 ± 53 mmHg versus 628 ± 123 mmHg in control animals (*p* = 0.02).

## 4. Discussion

In the current experiment, prestrike administration of verapamil did not prevent VF in a well-established commotio cordis model. Thus, intracellular calcium overload caused by activation of the I_Ca,L_ channel does not appear to be involved in the mechanism of VF in commotio cordis. Yet, intracellular calcium overload may contribute to the ST elevation and BBB observed in clinical cases and experimental models of chest wall trauma [3, 12].

Mechanical stimulation of the myocardium resulting in electrical events is well described, occurring in such circumstances as catheter-induced ectopy during intracardiac procedures and in the production of premature ventricular depolarizations by thumping the chest during asystole [13]. This phenomenon, termed mechanoelectric coupling, has been attributed to the presence of mechanosensitive ion channels that are activated by stretch or pressure changes within the myocardium. We have hypothesized that the inciting event in commotio cordis is the activation of mechanosensitive ion channels as a result of the rapid rise in left ventricular pressure and ensuing myocardial stretch [14]. We have previously shown that the initiation of VF in commotio cordis is related to the peak left ventricular pressure generated by the chest wall blow [3–6]. However, the cellular mechanisms that underlie the initiation of VF in commotio cordis remain incompletely understood and the identification of the potential mechanosensitive ion channels remains unresolved.

In ventricular myocytes, the L-type calcium current regulates excitation contraction coupling and influences the plateau height of the action potential, as well as total action potential duration [15]. During the plateau phase of the action potential, extracellular calcium enters the myocyte through the L-type calcium channel and elicits calcium release from the sarcoplasmic reticulum in a process termed calcium-induced calcium release [16, 17]. However, an excess of intracellular calcium may lead to EADs, DADs, and VF. Excessive intracellular calcium is the hallmark of several arrhythmogenic diseases caused by mutations in calcium regulatory genes.

Catecholaminergic polymorphic ventricular tachycardia is an arrhythmogenic disorder caused by mutations in one of the calcium regulatory genes [16]. Dominant mutations in the ryanodine receptor gene and recessive mutations in the calsequestrin-2 gene result in increased calcium current leak from the sarcoplasmic reticulum through the ryanodine receptor, resulting in intracellular calcium overload [17]. In CPVT, exercise or stress results in stimulation of beta adrenergic receptors, promoting cyclic AMP mediated phosphorylation of ryanodine receptors, leading to further intracellular calcium overload. The resultant excess calcium current can depolarize the myocyte at the end of the action potential, creating DADs. Delayed afterdepolarization reaching a critical threshold can trigger clinical arrhythmias, including ventricular extrasystoles, bidirectional ventricular tachycardia, and VF [17]. The mainstay of medical therapy for CPVT has been beta blockade, but there is evidence that treatment with verapamil may result in additional benefit [18]. It has been theorized that the effects of verapamil in CPVT are due to a reduction in the primary calcium signal through blockade of the L-type calcium channel [18, 19]. Verapamil might further reduce the calcium current through the ryanodine channel by indirectly reducing cyclic AMP or possibly by directly binding and blocking the channel [18, 20].

In Timothy syndrome, a “gain of function” mutation in the L-type calcium channel leads to reduced voltage dependent calcium channel inactivation [10]. The resulting inward calcium currents induce intracellular calcium overload that results in action potential prolongation and is associated with onset of DADs and triggered activity. Cardiac manifestations of Timothy syndrome include QT prolongation, torsades de pointes, and VF [10]. Blockade of the L-type calcium channel with verapamil can decrease the incidence of ventricular tachyarrhythmias in Timothy syndrome [21].

Calcium channels are known to exhibit mechanosensitive properties, including the cation nonselective stretch activated channel (SAC) and the more selective L-type calcium channels [8]. Mechanical stretch can result in increased intracellular calcium concentrations by direct activation of these channels, which further triggers the release of stored calcium in the sarcoplasmic reticulum [8]. In this study, administration of verapamil did not have a significant effect on the induction of VF in a commotio cordis model. The observation of a nonsignificant increased frequency of VF induction in the verapamil treated group likely relates to the higher maximum left ventricular pressures achieved from chest wall blows in this group. Although verapamil can have potent antifibrillatory effects in ischemia models [11, 22–25], presumably by decreasing myocardial ischemia, the instantaneous nature of VF in commotio cordis argues against ischemia as a cause of VF. Interestingly, verapamil pretreatment in our model did result in diminution of ST elevation and reduction in bundle branch block from chest wall impacts, suggesting that these effects may be mediated via stretch induced I_Ca,L_ channel activation.

The SAC has been shown to be activated by stretch in Langendorff models [26, 27] causing intracellular calcium increase and thus could also perhaps be involved in commotio cordis. However, a previous experiment in our model that targeted blockade of the SAC calcium channel did not show a reduction in the initiation of VF [28]. Interestingly, similar to verapamil pretreatment, blockade of SAC reduced the frequency and magnitude of ST elevation compared with controls. Taken in summation, these data suggest that although calcium channels, including SAC and the L-type calcium channel, exhibit mechanosensitive properties and are activated by forceful chest impact, they do not appear to play an integral role in the initiation of chest blow induced VF.

Despite the negative results with regard to VF shown in this current study and the previous SAC study, we do believe that commotio cordis is caused by ion channel activation produced by mechanical stretch of the cell membrane. The mechanosensitive K^+^_ATP_ channel has been identified among the ion channels activated by chest wall blow in commotio cordis [7]. We have previously shown that the infusion of glibenclamide, a sulfonylurea that acts primarily by inhibiting the K^+^_ATP_ channel, reduced the magnitude of ST elevation and the incidence of VF in our model. Further supporting the role of mechanoelectric coupling as the inciting event in commotio cordis are data revealing that initiation of VF by chest blows is significantly increased by selective disruption of the cytoskeleton [14]. These data suggest that mechanical deformation of the cell membrane is fundamental to the activation of ion channels and underlies the mechanism of VF in commotio cordis.

## 5. Limitations

Plasma verapamil levels were not measured; however, the dosing administered in this study was previously used in other experiments in whole animals of similar size [11]. Verapamil also inhibits vascular smooth muscle contraction and can result in vasodilation and hypotension. This is the likely reason that two of the animals given verapamil were not able to be resuscitated after successful defibrillation. The failure to resuscitate these animals reduced the number of impacts in swine, but we do not feel that this materially would have altered the outcome of this study. Even if all additional impacts in these animals failed to induce VF there would remain no significant difference in VF initiated by chest blows. In addition, we limited the velocity of impact to a single velocity of 40 mph, based on our studies that show that this is the velocity most likely to initiate VF in our experimental model [6].

## 6. Conclusion

In this study, infusion of verapamil did not alter the frequency of VF induction in our commotio cordis model. Verapamil did reduce the amount of ST elevation and the frequency of bundle branch block following chest impact. Our data indicate that although L-type calcium channels may exhibit mechanosensitive properties and may be activated by forceful chest wall impact, they do not play an integral role in the initiation of VF in commotio cordis. It is unlikely that myocardial intracellular calcium overload plays a vital role in the induction and maintenance of chest blow induced VF.

## Abbreviations

VF: Ventricular fibrillation
LV: Left ventricular
CPVT: Catecholaminergic polymorphic ventricular tachycardia
LQTS: Long QT syndrome
EAD: Early-after-depolarization
DAD: Delayed-after-depolarization
BBB: Bundle branch block.
